# Prognostic Significance of the Loss of Heterozygosity of *KRAS* in Early-Stage Lung Adenocarcinoma

**DOI:** 10.3389/fonc.2022.873532

**Published:** 2022-04-29

**Authors:** Anand Khadse, Vilde D. Haakensen, Laxmi Silwal-Pandit, Julian Hamfjord, Patrick Micke, Johan Botling, Odd Terje Brustugun, Ole Christian Lingjærde, Åslaug Helland, Elin H. Kure

**Affiliations:** ^1^Department of Cancer Genetics, Institute for Cancer Research, Oslo University Hospital, Oslo, Norway; ^2^Faculty of Technology, Natural Sciences and Maritime Sciences, Department of Natural Sciences and Environmental Health, University of South-Eastern Norway, Bø i Telemark, Norway; ^3^Department of Oncology, Oslo University Hospital, Oslo, Norway; ^4^Department of Immunology, Genetics and Pathology, Science for Life Laboratory, Uppsala University, Uppsala, Sweden; ^5^Section of Oncology, Drammen Hospital, Vestre Viken Hospital Trust, Drammen, Norway; ^6^Centre for Bioinformatics, Department of Informatics, University of Oslo, Oslo, Norway; ^7^Department of Clinical Medicine, University of Oslo, Oslo, Norway

**Keywords:** *KRAS*, LOH, prognostic marker, copy number aberration, NSCLC

## Abstract

Lung cancer is a common disease with a poor prognosis. Genomic alterations involving the *KRAS* gene are common in lung carcinomas, although much is unknown about how different mutations, deletions, and expressions influence the disease course. The first approval of a *KRAS*-directed inhibitor was recently approved by the FDA. Mutations in the *KRAS* gene have been associated with poor prognosis for lung adenocarcinomas, but implications of the loss of heterozygosity (LOH) of *KRAS* have not been investigated. In this study, we have assessed the LOH of *KRAS* in early-stage lung adenocarcinoma by analyzing DNA copy number profiles and have investigated the effect on patient outcome in association with mRNA expression and somatic hotspot mutations. *KRAS* mutation was present in 36% of cases and was associated with elevated mRNA expression. LOH in *KRAS* was associated with a favorable prognosis, more prominently in *KRAS* mutated than in wild-type patients. The presence of both LOH and mutation in *KRAS* conferred a better prognosis than *KRAS* mutation alone. For wild-type tumors, no difference in prognosis was observed between patients with and without LOH in *KRAS*. Our study indicates that LOH in *KRAS* is an independent prognostic factor that may refine the existing prognostic groups of lung adenocarcinomas.

## Introduction

Lung cancer is the leading cause of cancer-related deaths, causing an estimated 1.8 million deaths worldwide in 2018 (1). The overall 5-year survival rate for lung cancer patients during the period 2015–2019 in Norway was 22.7% in men and 29.2% in women, respectively (2). Lung adenocarcinoma is the most common type of non-small cell lung cancer (NSCLC) and accounts for 46% of all lung cancers in men and 52% in women (3). Adenocarcinoma is the most common type of lung cancer in never-smokers regardless of their age (4). Chromosomal abnormalities are frequent events in lung cancer, and both mutations and copy number aberrations can be the main drivers of the disease (5–7). Specific patterns of copy number gains and losses have been associated with histological subtypes of lung carcinomas, with lung adenocarcinoma displaying relatively fewer copy number alterations than lung squamous cell carcinoma and indicated to be mutation-driven (8, 9).

Deregulation of the Ras pathway by an activating Kirsten rat sarcoma viral oncogene (*KRAS*) mutation, occurring in about 30% of lung adenocarcinoma patients, is a hallmark of NSCLC (10). Previous studies have identified *KRAS* and *TP53* mutations (46%) as early events in carcinogenesis in patients with early-stage lung adenocarcinoma (11, 12). *EGFR* mutations (14%) are more common in lung adenocarcinomas of patients who never smoked, and those who exhibit such mutations benefit from *EGFR* inhibitors. They are found mutually exclusive with *KRAS* mutations which are associated with significant tobacco exposure (13, 14). *KRAS* mutations were described as a negative prognostic marker in metastatic lung adenocarcinoma (12, 15); however, the results have been inconsistent for early-stage disease and are still debated (16–18). An independent prognostic impact of *KRAS* mutations has been difficult to establish in relation to confounding concurrent tobacco-associated mutations such as *TP53*, *STK11*, and *KEAP1* (19). In recent years, tyrosine kinase inhibitors targeting *ROS1* mutations and *ALK* translocations have been introduced as the standard of care. Multiple therapies targeting the alterations in the *RTK*/*RAS*/*RAF* and *AKT*/*PI3K* pathways have been in development. Amplifications in *MET*, *PI3KCA*, and *ERBB2* were also in focus (7, 20). *KRAS* mutations have recently emerged as a useful negative predictive biomarker, predicting when therapy is unlikely to work. Despite decades of research, mutations in *KRAS* have been difficult to target due to the lack of surface targets for binding and its high affinity for GTP (21). Yet, recent results indicate the clinical effect of a selective *KRAS*^G12C^ inhibitor in a subgroup of patients with locally advanced or metastatic NSCLC (22, 23). The discovery provides an opportunity to selectively target *KRAS*^G12C^ in patients.

Loss of heterozygosity (LOH) is frequently observed in NSCLC, more frequent in squamous cell carcinomas than adenocarcinomas (24, 25). Several studies have reported allelic loss of chromosome 12p where *KRAS* resides, and that a loss correlates with the presence of *KRAS* mutation in human lung tumors (26). However, these studies did not investigate the prognostic impact of LOH in *KRAS*. Here, we have analyzed copy number profiles along with transcriptomic and mutation data of early-stage lung adenocarcinomas and assessed the prognostic significance of LOH in *KRAS*.

## Materials and Methods

### Patient Cohort

Participants included in the study were patients with operable early-stage lung adenocarcinomas surgically resected at Oslo University Hospital (OUH) from 2006 to 2011 (*n* = 133) and Uppsala University Hospital from 1995 to 2005 (*n* = 100). The study was approved by the Regional Committees for Medical and Health Research Ethics (REC) - South East Norway (reference no. S-06402b). We confirmed that all methods were performed in accordance with the relevant guidelines and regulations. All patients received oral and written information about the project and signed a written consent before entering the study. Clinical data from medical journals including follow-up were available for all patients. The main characteristics of the patients included are listed in **Table 1**. Some patients from stage II and stage III received adjuvant chemotherapy following standard guidelines. Tumor tissue was dissected and snap-frozen in liquid nitrogen and stored at −80°C until DNA and RNA isolation as previously described (27, 28). Tumor cellularity was estimated using the Allele-Specific Copy number Analysis of Tumors v2.3 (ASCAT) algorithm, and the samples with estimated tumor cell fraction greater than 20% were retained in the analysis.

**Table 1:** Patient characteristics.

Clinical features	Oslo cohort	Uppsala cohort	Combined
**Patients included**	133	100	233
**Sex**			
Female	75 (56%)	56 (56%)	131 (56%)
Male	58 (44%)	44 (44%)	102 (44%)
**Age**			
Mean (min–max)	65.5 (39–84)	63 (47–83)	64.5 (39–84)
**pStage**			
I	78 (59%)	69 (66%)	147 (63%)
II	32 (24%)	19 (18%)	51 (22%)
III	23 (17%)	12 (11%)	35 (15%)
IV	0	0	
**ECOG**			
0	63 (47.4%)	60 (60.0%)	123 (52.8%)
1	36 (27.1%)	32 (32.0%)	68 (29.2%)
2	5 (3.8%)	6 (4.0%)	11 (4.7%)
3	2 (1.5%)	2 (2.0%)	4 (1.7%)
**Smoking status**			
Smoker	118 (88%)	88 (88%)	206 (88%)
Never smoked	15	12	27
***KRAS* mutation**			
Mutated	46 (36%)	39 (39%)	85 (36.5%)
Wild-type	83	61	144
NA	4	0	4
***EGFR* mutation**			
Mutated	17 (13%)	17 (17%)	34 (15%)
Wild-type	115	83	198
NA	1	0	1
**Molecular subtypes**			
TRU	65 (60%)	50 (50%)	115 (55%)
PP	23 (21%)	31 (31%)	54 (26%)
PI	21 (19%)	19 (19%)	40 (19%)
NA	24	0	24
**Follow-up time, months**			
Median follow-up (IQR)	118 (113–126)	134 (91–167)	119 (110–137)
**Survival, months**			
Median overall survival (IQR)	78 (38–115)	48 (17–94)	68 (26–110)

NA, not assessed; IQR, interquartile range.

### Mutation Data Acquisition

Genomic DNA was extracted from frozen tumor tissue using the Maxwell^®^ 16 DNA purification kit (Promega, Madison, WI, USA) for the Oslo cohort and the QIAamp DNA Mini Kit (Qiagen, Hilden, Germany) for the Uppsala cohort, following the standard manufacturer’s protocol. *EGFR* mutation analyses of exons 18–21 were performed by real-time PCR using the therascreen *EGFR* mutation kit (Qiagen, Hilden, Germany) for the Oslo cohort. For the Uppsala cohort, PCR amplification of *EGFR* exons 18–21 was performed using the GeneAmp 2700 PCR cycler (Applied Biosystems, Waltham, MA, USA) and the ready-to-use ABgene PCR Master Mix (Thermo Fisher Scientific, Waltham, MA, USA) to determine *EGFR* mutation status. The Oslo cohort was analyzed for *KRAS* mutations using the Wobble-enhanced ARMS (WE-ARMS) method (29), while pyrosequencing and the PyroMark Q24 *KRAS* Kit (Qiagen, Hilden, Germany) were used to detect mutations in *KRAS* codons 12/13 (exon 2) and 61 (exon 3) in the Uppsala cohort. Separate PCR reactions for codons 12/13 and 61 were performed on the GeneAmp 2700 PCR cycler (Applied Biosystems, Waltham, MA, USA) as described in the original article (9).

### Gene Expression Profiling

The gene expression microarray SurePrint G3 Human GE, 8 × 60K (Agilent Technologies, Santa Clara, CA, USA) data for the subset of the Oslo cohort (*n* = 110) with GEO accession number GSE66863 was published previously (28). The data were log2-transformed and normalized between arrays by using the 75th percentile method in GeneSpring GX v.12.1 analysis software (Agilent Technologies, Santa Clara, CA, USA). The mRNA expression array includes 42,066 unique probes, and 30,370 probes remained after filtering out probes with no gene annotation or available gene names. The mRNA expression data for the Uppsala cohort (*n* = 100) were available on GEO (accession GSE37745). The samples were analyzed using Affymetrix Human Genome U133 Plus 2.0 Array and normalized using the Robust Multiarray Average (RMA) method. The average gene expression value was calculated when a gene mapped to more than one probe at the array.

### Estimation of Allele-Specific Copy Numbers

In the Oslo cohort, genomic DNA was extracted from the frozen tumor tissue using the Maxwell^®^ 16 DNA purification kit following standard protocol. The DNA was hybridized to Affymetrix genome-wide human SNP 6.0 arrays following the manufacturer’s instructions (Affymetrix, Santa Clara, CA, USA) at AROS Applied Biotechnology A/S (Aarhus, Denmark). Raw signal intensities were extracted and quantile-normalized using the Affymetrix Power Tools (APT) software and the PennCNV software to obtain log-transformed total signal intensities (LogR) for all probes and B allele frequencies (BAF) for SNP probes. After adjusting LogR for GC-binding artifacts, the LogR and BAF values were used as input for the allele-specific segmentation of normalized raw data by ASPCF with penalty parameter gamma = 70 and the subsequent analysis with ASCAT (30). The result was an allele-specific copy number profile of each tumor as well as estimates of tumor ploidy and tumor cell fraction (cellularity). ASCAT profiles were successfully obtained for 133 samples in the Oslo cohort, and these were used in the subsequent downstream analyses. Tumor samples of 104 lung adenocarcinomas from the Uppsala cohort were analyzed to obtain copy number profiles (31). Affymetrix Gene Chip Human Mapping 250K Nsp I arrays were used according to the manufacturer’s directions for the genomic DNA extracted from fresh frozen lung cancer tissue. Copy number analysis was performed using the ASCAT pipeline described above with changing platform parameter to “Affy250k_nsp” and obtained the ASCAT profiles for 100 samples by excluding four metastatic tumor profiles.

An ASCAT profile provides a segmentation of the genome into regions of constant allele-specific copy numbers. The total copy number of a segment is the sum of the major and minor allele copy numbers. Genomic regions with a total copy number greater or smaller than the tumor ploidy were considered as gains (amplifications) and losses (deletions), respectively. The *ploidy-adjusted* total copy number of a genomic region is determined by subtracting tumor ploidy from the total copy number of the region and rounded to the nearest integer. The genomic region with *ploidy-adjusted* total copy number 1 or above (i.e., 1, 2, 3…) was assigned as gain, whereas the region with a negative *ploidy-adjusted* total copy number (i.e., −1, −2, −3…) was assigned as loss. The hg19 genomic coordinates of SNP probes given in the array annotation file (release 35) were used to map aberrant genomic regions to the gene coordinates obtained from refFlat annotations. The chromosomal regions where only one allele (major or minor) was present were identified as regions with LOH. For each tumor, the genome instability index (GII) is defined as the fraction of the genome with loss or gain; in practice, this is calculated as the fraction of probes within segments with loss or gains.

### Statistical Analyses

All statistical analyses were performed using R version 3.6.2. The Pearson correlation coefficient was used to estimate correlations, and the false discovery rate (FDR) was used to correct for multiple testing. Pearson’s Chi-squared test, Fisher’s exact test, *t*-test, or logistic regression was used when appropriate to test associations between different variables. Regions with a significant difference in gains or losses at a given position in two groups were determined using the two-proportion *z*-test implemented in the *prop.test* function.

#### Frequency Plot

Samples were divided into two groups based on LOH status (present or absent) in the *KRAS* region. The frequency of gain (or loss) at a given genomic position in a group was calculated as the proportion of samples in that group with the aberration. Frequencies of gains were plotted on the *y*-axis in a positive scale, while the frequencies of losses are plotted in a negative scale. Chromosome-wise genomic positions are plotted on the *x*-axis.

#### Molecular Subtype Assignment

Adenocarcinomas were classified as terminal respiratory unit (TRU, formerly bronchioid), proximal-proliferative (PP, formerly magnoid), or proximal-inflammatory (PI, formerly squamoid) using the previously published centroid classifiers for adenocarcinomas (32). The subtype predictor centroids of 506 genes were used and samples were assigned to the closest centroid subtype.

#### Genome-Wide Correlation Analysis

The Pearson correlation coefficient was calculated to estimate the correlation between the copy number alteration and mRNA expression in 197 samples for which both copy number and expression data were available. The allele-specific copy number values of the genes were compared against normalized mRNA expression data. The correlation coefficients and *p*-values were reported for the regions where significant association (adjusted *p* < 0.05) was found between the gene expression and copy number state. The functional enrichment analysis of correlating genes was carried out using the Database for Annotation, Visualization and Integrated Discovery (DAVID) Functional Annotation Tool v.6.8.

#### Survival Analysis

The Kaplan–Meier estimator was used for the visualization of survival curves, the Log-rank test was used for testing differences between survival curves, and Cox proportional hazards (PH) regression was used to model and investigate survival as a function of covariates. All analyses were performed using the R package *survival* version 3.1.8. Overall survival (OS) time was calculated from the date of surgery to the time of death or censoring. Relapse-free survival (RFS) time was calculated from the date of surgery to the date of recurrence of disease or censoring. Patients were censored as of March 2020. Factors predicting the outcomes were assessed using the Cox proportional hazards model. Multivariate Cox proportional hazards regression analyses (adjusted for age and tumor stage) were used to analyze the correlation between *KRAS* LOH and survival in lung adenocarcinoma patients.

## Results

### Association of KRAS LOH to Clinicopathological and Molecular Features

**Figure 1:**
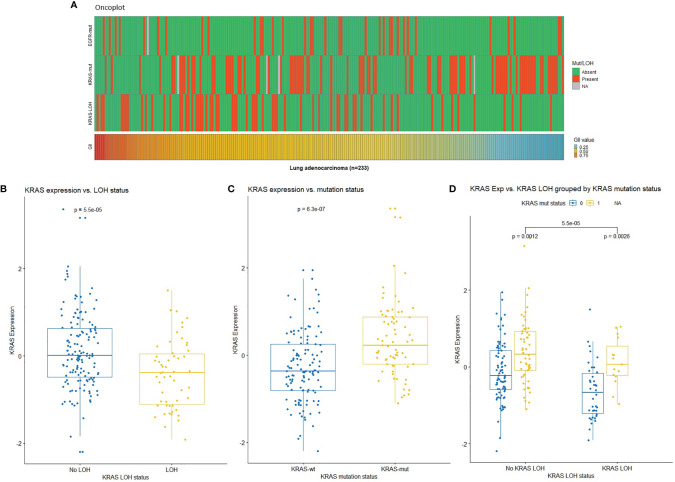
**(A)** Oncoplot showing mutation status in *EGFR* and *KRAS* (red: mutated, green: wild-type, gray: missing), loss of heterozygosity (LOH) at the *KRAS* locus (red: LOH, green: no LOH), and genomic instability index (GII) in the Oslo cohort (red: high, blue: low). **(B)** *KRAS* expression by *KRAS* LOH status indicating higher expression in samples with no LOH in *KRAS*. **(C)** *KRAS* expression by *KRAS* mutation status indicating high expression in samples with *KRAS* mutation (wt: wild-type; mut: mutation). **(D)** *KRAS* expression by *KRAS* LOH status grouped by *KRAS* mutation status in lung adenocarcinomas with respect to *KRAS* LOH and mutation status [0: wild-type (blue); 1: mutation (yellow)]. NA, Not available.

In order to investigate the possible genetic changes associated with LOH in *KRAS*, the tumors were further divided into two groups based on LOH at the *KRAS* locus (12p12.1). No significant association was observed between *KRAS* LOH and clinicopathological characteristics in this patient cohort (**Table 2**). Mutations and GII in the samples are illustrated in **Figure 1A**. There was no clear association between *KRAS* LOH and mutation (odds ratio = 0.51, Fisher’s exact *p* = 0.057). Tumors with LOH at the *KRAS* locus had an overall higher genomic instability than tumors without LOH (average GII of 0.57 compared to 0.46; *p* < 0.001, *t*-test), although high GII was not found to be associated with the overall survival [HR = 0.913; 95% confidence interval (CI) 0.427–1.951; *p* = 0.814].

**Table 2:** Association between *KRAS* LOH status and clinicopathological characteristics.

Clinical features	With LOH in *KRAS*	No LOH in *KRAS*	*χ*^2^ or Fisher’s exact test
	No. (%)	No. (%)	*p-*value
**Patients included**	62	171	
**Sex**			
Female	34 (55%)	97 (57%)	0.915
Male	28 (45%)	74 (43%)	
**Age** (years)			
<70	40 (70%)	113 (68%)	0.629
≥70	17 (30%)	54 (32%)	
**pStage**			
I	44 (71%)	103 (60.5%)	0.229
II	9 (14.5%)	42 (24.5%)	
III	9 (14.5%)	26 (15%)	
IV	0	0	
**Smoking status**			
Smoker	52 (84%)	154 (90%)	0.284
Never smoked	10	17	
***KRAS* mutation**			
Mutated	16 (26%)	69 (40%)	0.057
Wild-type	45	99	
NA	1	3	
***EGFR* mutation**			
Mutated	10 (16%)	24 (14%)	0.862
Wild-type	52	146	
NA	0	1	
**Molecular subtypes**			
TRU	34 (59%)	81 (54%)	0.806
PP	14 (24%)	40 (26%)	
PI	10 (17%)	30 (20%)	
NA	4	20	
**Overall survival, months**			
Median (Q1–Q3)	91 (46–118)	60 (22–108)	

### Genomic Aberrations in Early-Stage Lung Adenocarcinomas

The lung adenocarcinomas displayed overall complex DNA copy number profiles with recurrent aberrations in almost all chromosomes. Recurrent gains were observed on 1q, 5p, 6p, 7p, 8q, 14p, 17q, and 20p in more than 25% of cases, and similarly, recurrent losses on 3p, 5q, 6q, 8p, 9p, 9q, 10q, 13q, 15q, 17p, 18q, 19p, 21q, and 22q were observed in more than 25% of cases. LOH was observed in the *KRAS* gene in 26.6% of cases. Frequencies of genomic alterations, i.e., the percentage of patients with gains or losses across the genome, are shown for the complete lung adenocarcinoma cohort (*n* = 233) (**Figure S1A**) and in individual cohorts (**Figure S1B**) in **Supplementary Figure S1**. Hotspot mutation frequency of the total number of samples was 36.5% for *KRAS* and 14.6% for *EGFR*. *EGFR* and *KRAS* mutations were mutually exclusive. The most frequent *KRAS* mutations observed in the samples were G12C (37%), G12V (21%), and G12D (19%) (**Supplementary Figure S2**).

Significant differences in gains or losses across 15-kb genomic intervals were identified using *prop.test* (*p* < 0.05) by comparing the proportion of amplification or deletion at a given interval in the two groups. Chromosomes 1p, 7p/q, 8p, 12p, 13q, and 16q showed significant differences in gains between the two groups. Similarly, significant differences in losses were identified at 1p, 2q, 5p/q, 9p/q, 10q, 12 p/q, 13q, 15q, 17p/q, 19q, and Xp region (**Figure S1B**).

None of the samples had a complete loss of *KRAS*. Twelve samples had copy-neutral LOH in *KRAS*. These samples had loss of one *KRAS* allele, but the total copy number at the locus was equal to tumor ploidy. The expression of *KRAS* was lower in the samples with LOH at *KRAS* compared with samples without LOH (**Figure 1B**). We observed higher *KRAS* expression in *KRAS* mutated compared with wild-type samples (**Figure 1C**). When the samples were stratified by *KRAS* mutation status, mutant *KRAS* samples showed higher expression than wild-type *KRAS* samples in *KRAS* LOH-positive samples (**Figure 1D**).

### Prognostic Significance of Loss of Heterozygosity in *KRAS*

**Figure 2:**
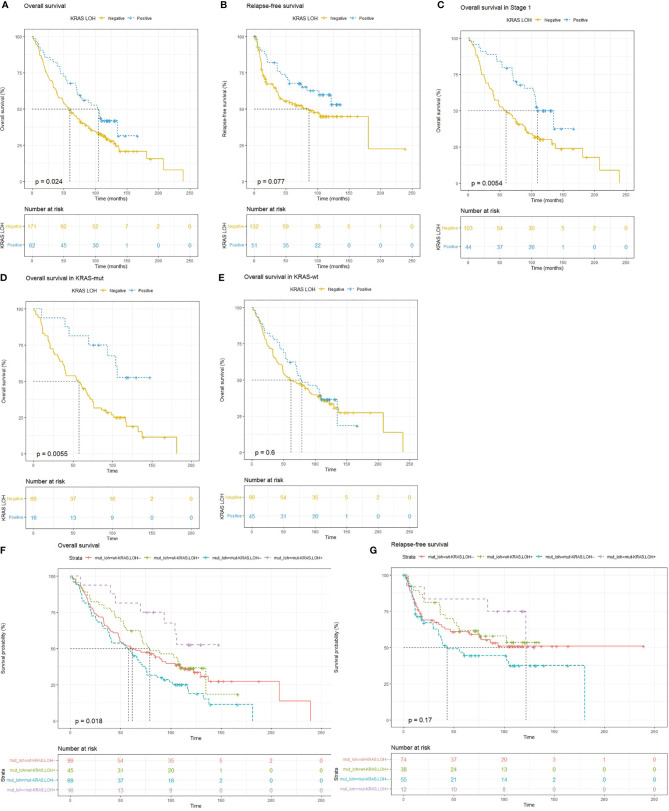
Kaplan–Meier survival plots for **(A)** overall survival (OS) and **(B)** relapse-free survival (RFS) in patients with lung adenocarcinomas with and without LOH in *KRAS* indicating improved survival with LOH in *KRAS*. **(C)** OS in patients with stage I disease. **(D)** OS in wild-type *KRAS* tumors and **(E)** *KRAS* mutated tumors. **(F, G)** OS and RFS in patients based on combined *KRAS* mutation and LOH status, where patients with both *KRAS* mutation and *KRAS* LOH (purple) have better OS and RFS, whereas patients with only *KRAS* mutation and no *KRAS* LOH (blue) have the worst OS as well as RFS.

Lung adenocarcinoma patients with LOH in *KRAS* had significantly better OS (HR = 0.65; 95% CI 0.45–0.95; *p* = 0.025) (**Figure 2A**) compared with patients with no *KRAS* LOH. A similar trend was also seen in RFS although statistical significance was not reached (HR = 0.64; 95% CI 0.39–1.06; *p* = 0.08) (**Figure 2B**). Multivariate analyses also suggest a tendency of improved OS for patients with LOH in *KRAS* compared with no LOH (HR = 0.69; 95% CI 0.46–1.06; *p* = 0.09) (**Table 3**).

**Table 3:** Univariate and multivariate Cox proportional hazards regression analyses of different prognostic variables for overall survival (OS) and relapse-free survival (RFS) in early-stage lung adenocarcinoma.

Factors	Overall survival (OS)				Relapse-free survival (RFS)			
	Univariate analysis		Multivariate analysis		Univariate analysis		Multivariate analysis	
	HR (95% CI)	*p*-value	HR (95% CI)	*p*-value	HR (95% CI)	*p*-value	HR (95% CI)	*p*-value
Age	1.031 (1.013–1.05)	**<0.001**	1.033 (1.013–1.054)	**0.001**	1.003 (0.978–1.029)	0.787	1.007 (0.979–1.036)	0.624
Sex (male vs. female)	1.044 (0.763–1.427)	0.788	NA		0.988 (0.639–1.529)	0.958	NA	
Stage (II + III vs. I)	1.327 (0.965–1.823)	0.081	1.305 (0.898–1.897)	0.162	2.121 (1.377–3.267)	**<0.001**	1.959 (1.204-3.188)	**0.007**
ECOG (1 + 2 + 3 vs. 0)	1.266 (0.907–1.765)	0.165	NA		0.701 (0.426–1.152)	0.161	NA	
Genome instability index (high vs. low)	0.913 (0.427–1.951)	0.814	NA		1.456 (0.524–4.045)	0.471	NA	
*EGFR* mutation (present vs. absent)	0.915 (0.583–1.436)	0.698	NA		0.787 (0.406–1.526)	0.478	NA	
*KRAS* expression	1.106 (0.905–1.335)	0.297	0.987 (0.792–1.230)	0.908	1.212 (0.953–1.541)	0.118	1.017 (0.771–1.342)	0.903
*KRAS* mutation (present vs. absent)	1.224 (0.889–1.685)	0.214	1.316 (0.892–1.940)	0.165	1.261 (0.812–1.957)	0.302	1.324 (0.791–2.218)	0.285
*KRAS* LOH (present vs. absent)	0.653 (0.449–0.948)	**0.025**	0.694 (0.456–1.056)	**0.088**	0.637 (0.385–1.055)	**0.08**	0.705 (0.398–1.249)	0.230

Statistically significant p-values in bold. NA, Not applicable.

In order to adjust the effect of any potential confounder on survival associated with LOH in *KRAS*, we performed bivariate Cox proportional hazards regression analyses with LOH in *KRAS* as a fixed independent variable including potential covariate in the bivariate model (**Supplementary Table S1**). The analysis showed that the effect of LOH in *KRAS* on OS as well as on RFS was unaffected even after adjusting the effect of confounders such as genomic instability and *KRAS* mutations, suggesting LOH in *KRAS* as an independent prognostic factor.

#### *KRAS* LOH in Stage I Lung Adenocarcinomas

The Cox proportional hazards regression model for patients with LOH in *KRAS* adjusted for progression stage suggested reduced hazard for OS [HR = 0.682 (0.46–0.99), *p* = 0.04]. The difference in the fraction of samples with *KRAS* LOH in different stages can be observed in **Table 2**. In stage I, a greater number of samples had LOH in *KRAS*. To remove the stage bias, we assessed the effect of *KRAS* LOH in stage I patients separately. The Kaplan–Meier estimator showed that patients with stage I disease and LOH at *KRAS* had better OS (*p* = 0.005) than patients with no *KRAS* LOH. The median OS in the patients with LOH in *KRAS* was 9 years, compared with 5 years in the patients with both alleles intact (**Figure 2C**).

#### *KRAS* LOH According to *KRAS* Mutation Status

To test whether LOH in the *KRAS* locus influenced prognosis in *KRAS* mutated patients, we performed Kaplan–Meier analysis on the patients with *KRAS* mutation and patients with wild-type *KRAS* separately. In the subgroup of patients with *KRAS* mutation, we found that LOH in *KRAS* conferred better OS (*p* < 0.01) compared with no LOH. No such difference in survival was observed in patients with wild-type *KRAS* (**Figures 2D, E**). We further evaluated the combined effect of *KRAS* LOH and *KRAS* mutation on overall survival and recurrence-free survival of the patients (**Figures 2F, G**). Kaplan–Meier curves suggest a significantly shorter survival time in patients with mutated *KRAS* without *KRAS* LOH, whereas patients with mutated *KRAS* with LOH have the most favorable OS as well as RFS.

### Alterations in Hotspots

**Figure 3:**
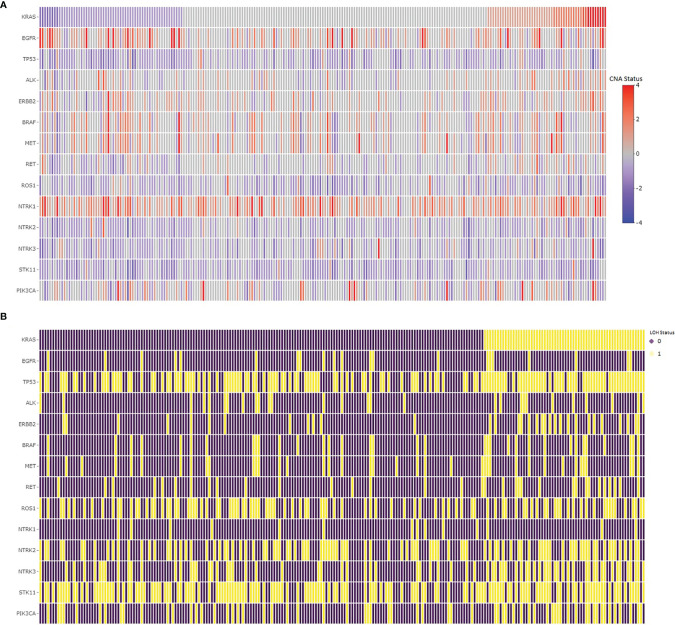
Copy number changes **(A)** and LOH **(B)** in common hotspots in lung adenocarcinomas. **(A)** The copy number changes in the hotspot gene regions sorted by copy number changes in the *KRAS* gene, where the red color represents copy number gain, whereas the blue color represents copy number loss in the regions. **(B)** The loss of heterozygosity in the hotspot gene regions, where the purple color shows no loss, whereas the yellow color represents loss of heterozygosity in the region.

We assessed common hotspot mutation regions for copy number alterations in lung adenocarcinomas to determine their concurrences and associations with LOH in *KRAS*. The copy number changes and the LOH statistics for *EGFR*, *TP53*, *ALK*, *ERBB2*, *BRAF*, *MET*, *RET*, *ROS1*, *HER2*, *NTRK*, *STK11*, and *PIK3CA* are given in **Table 4**. The copy number changes and the LOH in common hotspot gene regions relative to *KRAS* in lung adenocarcinoma samples are shown in **Figure 3**. The chi-square test shows the LOH in *TP53*, *ERBB2*, *BRAF*, *RET*, *NTRK3*, and *PIK3CA* associated with LOH in *KRAS*. Sixty percent of the samples had *TP53* copy number loss and 85% of the *KRAS* LOH samples also had LOH in *TP53* with statistically significant association. Similarly, *STK11* had a high percentage (64%) of LOH in the samples, but they are not associated with LOH in *KRAS*.

**Table 4:** Copy number changes in common hotspots in lung adenocarcinomas and associations between LOH in genetic hotspot regions and *KRAS* LOH.

Regions	Gain (%)	Loss (%)	LOH (%)	*KRAS* LOH samples	Association with *KRAS* LOH	
				(LOH %)	(*χ*^2^ *p*-value)	FDR-adjusted *p*-value
*KRAS*	21.03	25.32	26.61			
*EGFR*	45.49	8.58	9.01	11.29	0.6368	0.6899
*TP53*	4.72	59.66	60.52	85.48	**5.54E−06**	**3.60E−05**
*ALK*	18.45	12.88	12.02	17.74	0.1645	0.2672
*ERBB2*	29.18	11.59	13.73	32.26	**2.23E−06**	**2.90E−05**
*BRAF*	31.33	13.73	14.59	24.19	0.02203	**0.04774**
*MET*	31.76	14.59	16.74	22.58	0.215	0.3046
*RET*	12.88	24.89	14.16	25.81	**0.004281**	**0.01112**
*ROS1*	5.15	51.07	42.06	46.77	0.4669	0.5517
*NTRK1*	65.67	1.29	6.01	4.84	0.8882	0.8821
*NTRK2*	3.86	53.22	42.92	53.23	0.07768	0.1144
*NTRK3*	6.87	46.78	31.76	54.84	**1.10E−05**	**4.75E−05**
*STK11*	2.15	66.52	63.95	70.97	0.2343	0.3046
*PIK3CA*	21.46	26.61	27.04	43.55	**0.001156**	**3.76E−03**

Statistically significant p-values in bold.

### Correlating Genes and Association With Survival

Somatic copy number changes may contribute to the development and progression of cancer by affecting gene expression level (33). Improved knowledge about genomic alterations and expression profiles in the subgroups defined by *KRAS* LOH may identify genes involved in disease development or outcome. We performed a genome-wide correlation analysis to identify genomic aberrations associated with *KRAS* LOH with an effect on gene expression. We found the mRNA expressions of 9,663 genes to be significantly correlated with *KRAS* LOH (adjusted *p* < 0.05), of which 2,474 genes had a moderately high correlation (*r* > 0.4) to their ploidy-adjusted copy number state. Correlation analysis showed a moderate correlation (*r* = 0.4) between *KRAS* expression and its copy number in the genome. The detailed list of the significantly correlated genes is given in **Supplementary Table S2**. From the 2,474 significantly correlated genes, we found 1,371 deleted genes, while 852 genes showed significant gains in copy number in the samples with LOH in *KRAS*. Functional analysis using DAVID showed that genes with copy number losses were involved in biological processes such as alternate splicing, protein transport, ubiquitin-mediated proteolysis, cell cycle, and DNA repair. Genes with gains in copy number were enriched for genes involved in transcription regulation, ribosomes, and mRNA processing. This overview of the genomic and transcriptional landscape in lung adenocarcinoma stratified by LOH in *KRAS* suggests that the overall genomic and transcriptional landscape of lung adenocarcinoma is affected to some extent by the *KRAS* LOH status.

We further conducted differential expression analysis between samples with and without *KRAS* LOH and identified 197 genes that were significantly differentially expressed in both cohorts. From the differentially expressed genes, 38 genes were overexpressed, of which 15 genes were significantly amplified, and 82 genes were underexpressed, of which 70 were significantly deleted in the samples with *KRAS* LOH. Significantly amplified and deleted gene regions with significant correlation for their mRNA–copy number in samples with LOH in *KRAS* with respect to no LOH samples are listed in **Supplementary Table S3**. Gene set enrichment analysis of these genes shows their role in RNA transport pathways and protein-binding function (**Supplementary Table S4**).

Eight of the amplified genes and 66 of the deleted genes showed a significant correlation between their copy number and expression. We investigated the association of these genes to patient outcome and found that overexpression of *CDC14A* and downregulation of *GABARAPL1* and *RFK* in the *KRAS* LOH-positive samples were significantly associated with OS. The survival analysis showed that high *CDC14A* expression was significantly associated with improved OS. Similarly, the lower expression of *GABARAPL1* and *RFK* was associated with improved OS in lung adenocarcinoma patients (**Supplementary Figure S3**). The genes functionally separate the two subgroups and may be involved in the outcome.

## Discussion

In this study, we identified the number of recurrent genomic aberrations in a lung adenocarcinoma subgroup defined by *KRAS* LOH status. The study demonstrates that for patients with early-stage lung adenocarcinomas, loss of heterozygosity in the *KRAS* region is associated with improved OS as well as RFS. The genome-wide copy number analysis showed gains and losses consistent with the results reported in previously published studies (34, 35). In order to identify the genes whose mRNA expression had changed due to change in copy number, we performed a correlation analysis between copy number and mRNA expression data and found 2,474 genes with statistically significant correlation (*r* > 0.4, *p*.adjust < 0.05). We found relatively few genes with correlation between mRNA expression and copy number compared to a previously published study (31). *KRAS* gene expression showed a moderately high correlation to its copy number, which signifies the importance of copy number analysis in relation to *KRAS*.

We identified significant differences in aberration pattern between patients with and without *KRAS* LOH. To our knowledge, no previous studies have investigated the aberration pattern of tumor DNA based on LOH in the *KRAS* region. Survival analysis showed that LOH in the *KRAS* region correlates with survival and is associated with improved prognosis more specifically in patients with *KRAS* mutated tumors. We tested other clinicopathological parameters for their association with *KRAS* LOH and found no significant association, suggesting *KRAS* LOH as an independent prognostic factor. Previous studies found *KRAS*^G12C^ mutations associated with negative clinical outcomes in advanced cancers. The recent finding of the selective *KRAS*^G12C^ inhibitor (23) shows that *KRAS* is no longer undruggable. This shows the importance of molecular characterization of the tumor of each patient diagnosed with lung cancer to identify druggable targets. Our study identified an improved OS in *KRAS* mutated tumors with LOH in *KRAS*, irrespective of which hotspot *KRAS* mutation was present. We also found that a relatively small number of *KRAS* mutated tumors had LOH in the gene, with a slightly negative correlation for their co-occurrence.

Univariate analysis using Cox proportional hazards regression showed that only age, stage, and *KRAS* LOH were the significant factors for OS. Multivariate Cox proportional hazards regression analysis showed that *KRAS* LOH has a weak significance as a prognostic factor for OS (*p*-value = 0.08), but not for RFS. LOH in *KRAS* was a statistically significant prognostic factor in stage I disease with mutant *KRAS*. The mRNA expression and copy number state of *KRAS* have a moderately high correlation, and the loss of an allele in *KRAS* resulted in a lower overall expression of the gene. While LOH in *KRAS* is associated with reduced expression of the *KRAS* gene in *KRAS* wild-type samples, this is not the case in the *KRAS* mutated samples. This could indicate that mutant *KRAS* with LOH leads to an always-ON state of the gene, and the interaction with wild-type *KRAS* in no LOH samples may be regulating the expression of mutant *KRAS.* This phenomenon may be attributed to the repressive effect of the wild-type allele in the *KRAS* mutated sample when both alleles are present (36, 37). This indicates that the association between LOH in *KRAS* and survival is not explained by the expression of the *KRAS* gene.

Hotspot mutations in lung cancer are well characterized for their concurrences (38). We analyzed copy number changes in these hotspot genes to determine their concurrences and associations with LOH in *KRAS*. *TP53* mutations are known to be associated with smoking and *KRAS* mutation. We found LOH in *TP53* to be associated with LOH in *KRAS*. We also found that mutation in the hotspot genes such as *RET*, *NTRK3*, and *PIK3CA* regions is associated with LOH in *KRAS*. We did integrative analyses to investigate the differential gene expression and copy number changes in the samples with *KRAS* LOH compared with those without *KRAS* LOH. We found 66 genes deleted with lowered mRNA expression, while 10 genes were amplified along with an increase in their mRNA expression levels in the samples with *KRAS* LOH. We found that the *CDC14A* gene that plays a role in cell cycle regulation was amplified along with increased mRNA expression level in *KRAS* LOH-positive samples, and its expression was positively associated with OS. Previous studies suggested that the gene was differentially expressed in cancers and it can interact with the tumor suppressor p53 (39, 40). An autophagy-related gene *GABARAPL1* was found deleted with lowered expression in the samples with *KRAS* LOH. Studies have shown that its expression is associated with better outcome in breast cancers (41). In contrast, we found the *lower* expression of *GABARAPL1* to be associated with better outcome in our study. We believe that these genetic differences can provide new approaches to refine prognostication of lung adenocarcinomas and deserve to be explored further.

In conclusion, our study shows that LOH in *KRAS* is associated with a favorable prognosis in patients with early-stage lung adenocarcinomas, particularly in patients with *KRAS* mutated tumors. Our study indicates that LOH in *KRAS* is a prognostic factor that can refine the existing prognostic groups of lung adenocarcinomas.

## Data Availability Statement

The original contributions presented in the study are included in the article/**Supplementary Material**. Further inquiries can be directed to the corresponding author.

## Ethics Statement

The studies involving human participants were reviewed and approved by the Regional Committees for Medical and Health Research Ethics Norway (ref: S-06402b). The patients/participants provided their written informed consent to participate in this study.

## Author Contributions

AK, VDH, OCL, JH, and EHK designed the study. AK performed the bioinformatics analyses with the help of LS-P and OCL. OTB, PM, JB, and ÅH provided the clinical data. AK and VDH wrote the first draft of the manuscript. EHK and ÅH supervised the study and were responsible for the funding. All authors critically reviewed the manuscript.

## Funding

This research was supported by the University of South-Eastern Norway and Institute for Cancer Research, Oslo University Hospital, Oslo, Norway, Grant Number: 88503-2013, the Norwegian Cancer Society.

## Conflict of Interest

The authors declare that the research was conducted in the absence of any commercial or financial relationships that could be construed as a potential conflict of interest.

## Publisher’s Note

All claims expressed in this article are solely those of the authors and do not necessarily represent those of their affiliated organizations, or those of the publisher, the editors and the reviewers. Any product that may be evaluated in this article, or claim that may be made by its manufacturer, is not guaranteed or endorsed by the publisher.
