# Supplementary material for: Prognostic Significance of the Loss of Heterozygosity of KRAS in Early-Stage Lung Adenocarcinoma
Source: Front Oncol. 2022 Apr 29;12:873532. doi: 10.3389/fonc.2022.873532 (PMC9098994; doi:10.3389/fonc.2022.873532)
Supplement: Supplementary file 1 [file DataSheet_1.docx]

A


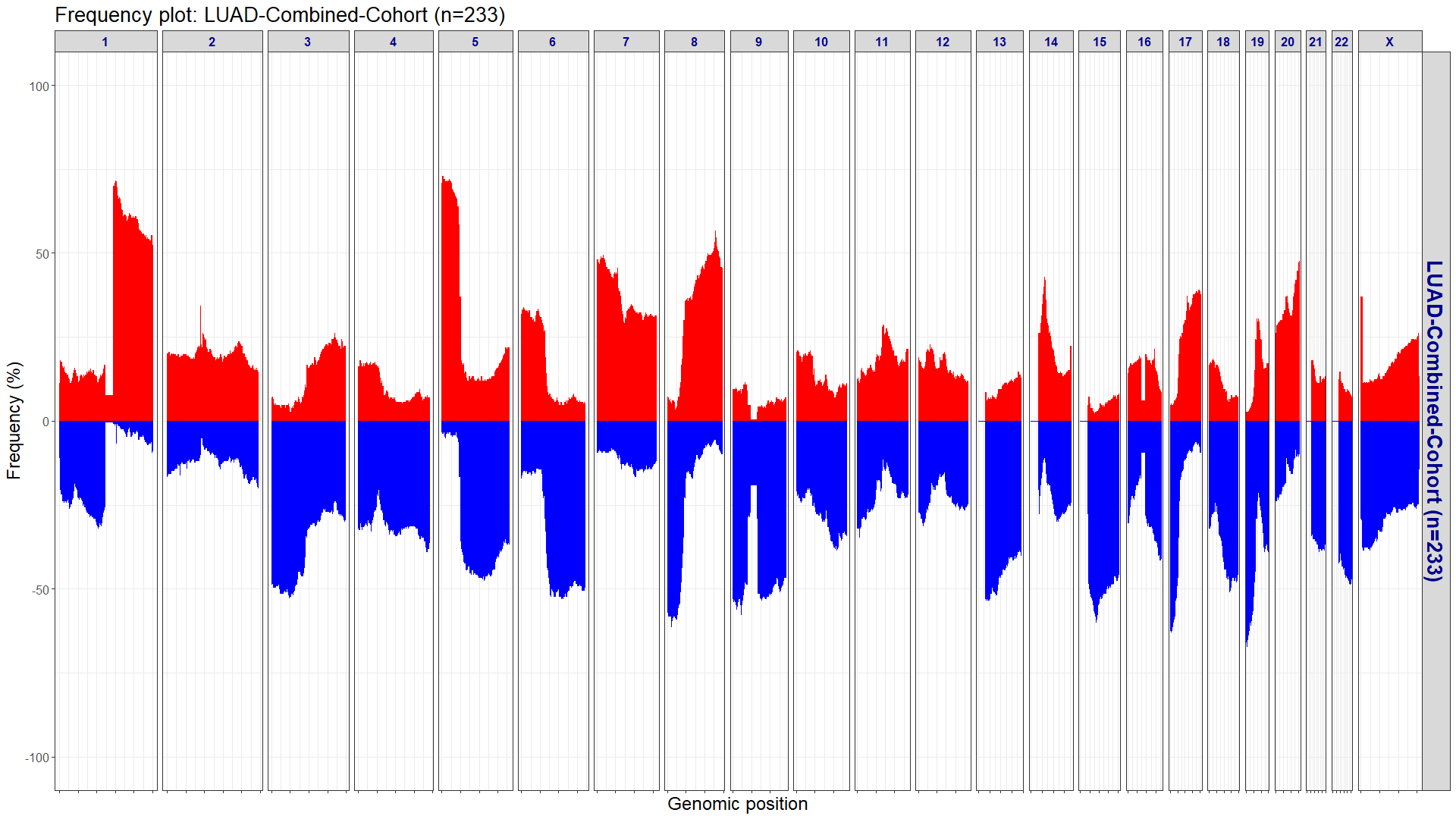


B


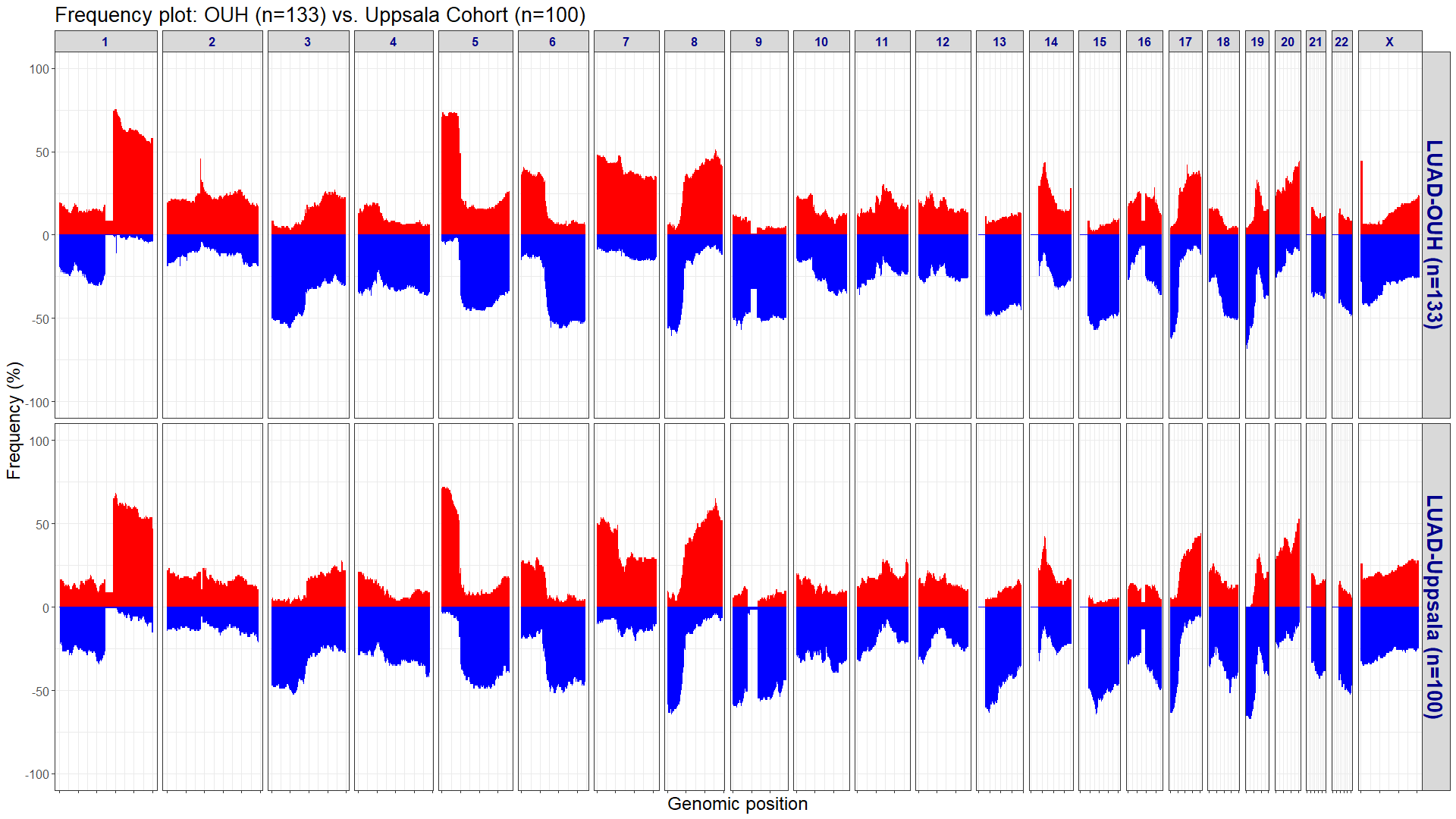


**Figure S1:** Frequency plots of copy number aberration (amplifications in red and deletions in blue), **(A)** in lung adenocarcinomas, **(B)** in tumors stratified on LOH status at the *KRAS* locus.


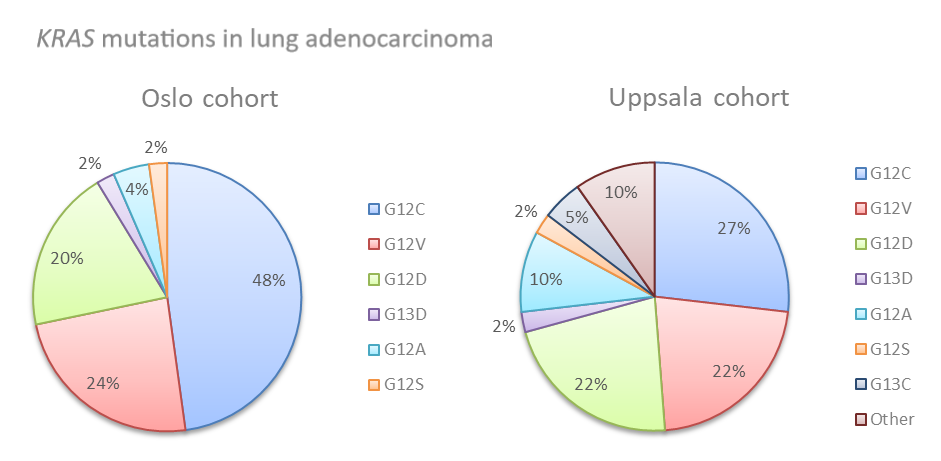
**Figure S2:** *KRAS* gene mutations in lung adenocarcinoma samples from the Oslo cohort and Uppsala cohort


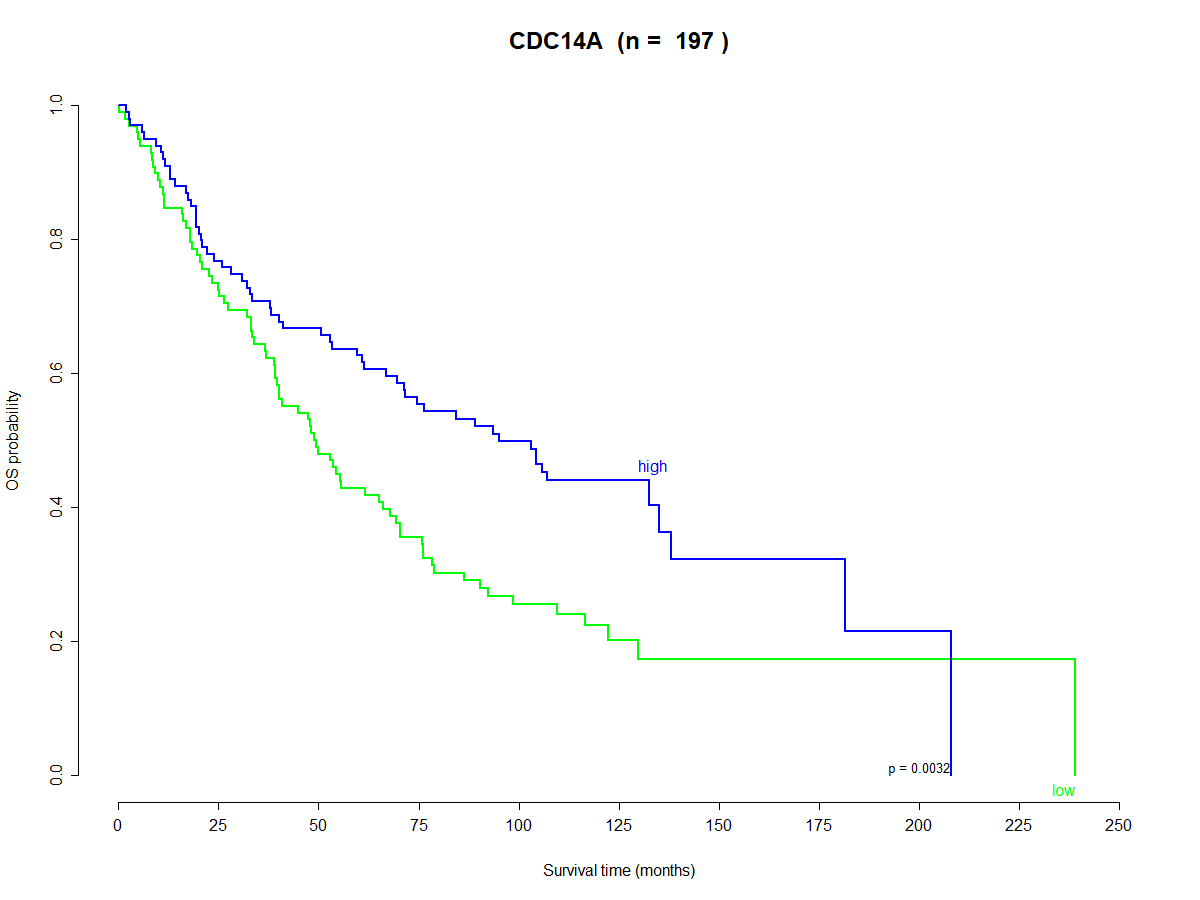

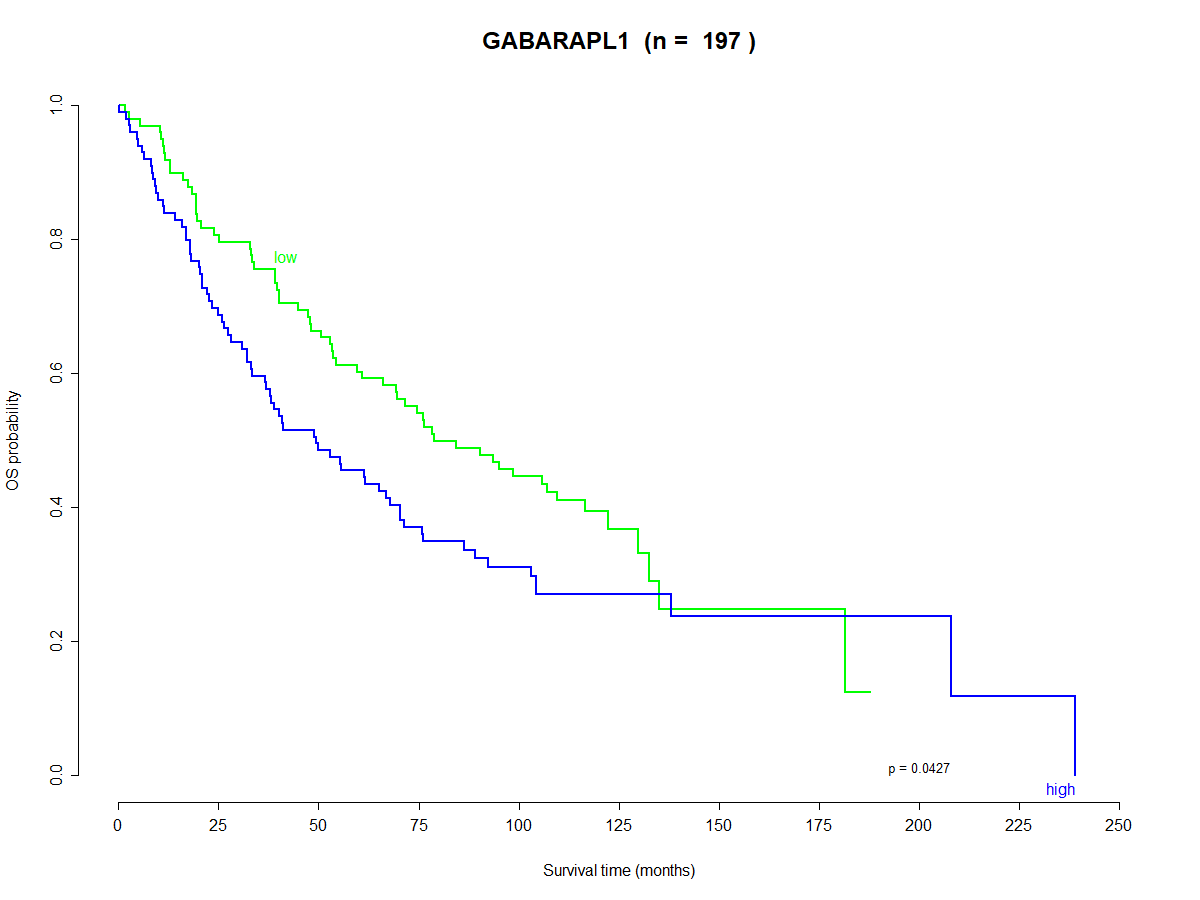

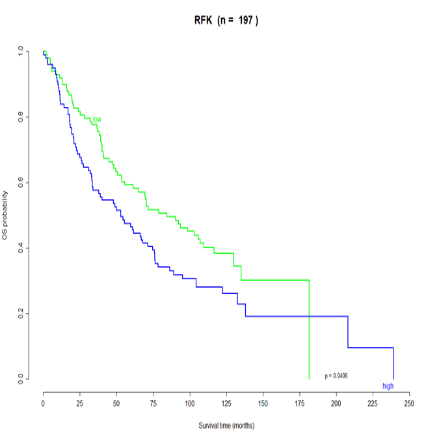


**Figure S3:** Kaplan-Meier survival curve for OS in lung adenocarcinoma for expression of *CDC14A, GABARAPL1* and *RFK*
